# Immunoprofile from tissue microarrays to stratify familial breast cancer patients

**DOI:** 10.18632/oncotarget.4720

**Published:** 2015-08-03

**Authors:** Laura Schirosi, Simona De Summa, Stefania Tommasi, Angelo Paradiso, Domenico Sambiasi, Ondina Popescu, Giovanni Simone, Anita Mangia

**Affiliations:** ^1^ Functional Biomorphology Laboratory, IRCCS Istituto Tumori “Giovanni Paolo II”, 70124 Bari, Italy; ^2^ Molecular Genetic Laboratory, IRCCS Istituto Tumori “Giovanni Paolo II”, 70124 Bari, Italy; ^3^ Experimental Medical Oncology, IRCCS Istituto Tumori “Giovanni Paolo II”, 70124 Bari, Italy; ^4^ Pathology Department, IRCCS Istituto Tumori “Giovanni Paolo II”, 70124 Bari, Italy

**Keywords:** immunoprofile, familial breast cancer, hierarchical clustering analysis, TMA

## Abstract

Familial breast cancer (BC) is a heterogeneous disease with variable prognosis. The identification of an immunoprofile is important to predict tumor behavior for the routine clinical management of familial BC patients. Using immunohistochemistry on tissue microarrays, we studied 95 familial BCs in order to analyze the expression of some biomarkers involved in different pathways. We used unsupervised hierarchical clustering analyses (HCA), performed using the immunohistochemical score data, to define an immunoprofile able to characterize these tumors. The analyses on 95 and then on a subset of 45 tumors with all biomarkers contemporarily evaluable, revealed the same biomarker and patient clusters. Focusing on the 45 tumors we identified a group of patients characterized by the low expression of estrogen receptor (*P* = 0.009), progesterone receptor (*P* < 0.001), BRCA1 (*P* = 0.005), nuclear Na^+^/H^+^ exchanger regulatory factor 1 (NHERF1) (*P* = 0.026) and hypoxia inducible factor-1 alpha (*P* < 0.001), and also by the higher expression of MIB1 (*P* = 0.043), cytoplasmic NHERF1 (*P* = 0.004), cytoplasmic BRCT-repeat inhibitor of hTERT expression (*P* = 0.001), vascular endothelial growth factor (VEGF) (*P* = 0.024) and VEGF receptor-1 (*P* = 0.029). This immunoprofile identified a more aggressive tumor phenotype associated also with a larger tumor size (*P* = 0.012) and G3 grade (*P* = 0.006), confirmed by univariate and multivariate analyses. In conclusion, the clinical application of HCA of immunohistochemical data could allow the assessment of prognostic biomarkers to be used simultaneously. The 10 protein expression panel might be used to identify the more aggressive tumor phenotype in familial BC and to direct patients towards a different clinical therapy.

## INTRODUCTION

Breast cancer (BC) is the most frequent malignant disease, and the leading cause of cancer death among women. It is currently estimated that approximately 5–10% of all BCs have a hereditary background. However, in patients with a suggestive personal and/or family history, a specific predisposing mutation in the breast cancer susceptibility gene-1 (*BRCA1*) or in the breast cancer susceptibility gene-2 (*BRCA2*) is identified in only fewer than 30% of cases [1]. Familial BC currently represents a heterogeneous disease, including different clinicopathological characteristics and different clinical behaviors. Therefore, it is of major importance to define the morphological, immunohistochemical, and molecular features of this group of tumors to gain further insight into their biological phenotype [2]. Although an exhaustible number of molecular studies on BC, almost all based on microarray profiling, have been published until now, few studies analyzing familial BC exist [1]. Microarray techniques, however, are rather expensive and not readily available, thus immunohistochemistry (IHC), for its lower costs and easy implementation into standard pathology workflow, could help to define protein biomarkers for the characterization of familial BC [3]. It has already been demonstrated that molecular classification by microarray analysis corresponds reasonably well to immunohistochemical classification of different breast carcinoma phenotypes [4, 5]. Breast tumors express some immunohistochemical markers providing both prognostic and predictive information; currently, estrogen receptor (ER), progesterone receptor (PR), proliferative activity and human epidermal growth factor receptor 2 (HER2) are among the most important ones, used for identifying poor prognosis breast tumors and for the selection of the most efficient therapies [6]. However, there is an urgency to identify different biomarkers involved in signaling pathways that govern the processes of formation, maintenance and spread of breast tumors in order to better stratify patients [7]. In particular, we have recently studied Na^+^/H^+^ exchanger regulatory factor 1 (NHERF1), a protein involved in transmitting signals from the surface into the cell, and we showed that the loss of nuclear NHERF1 expression in BC is associated with reduced survival and could represent a new valid prognostic marker [8]. Proteins involved in the mechanisms of DNA repair in cells, such as BRCA1 and Poly [ADP-ribose] polymerase 1 (PARP1), have also been studied in BC as markers to select patients for target therapy trials [9, 10] or for prognosis [11, 12]. The angiogenic pathway, on the other hand, is very important in tumor development and metastasis formation and this occurs also in breast tumors [7]. We have previously studied different angiogenic markers such as vascular endothelial growth factor (VEGF), hypoxia inducible factor-1 alpha (HIF-1α) and microvessel density (MVD) in familial BC and we suggested that angiogenesis plays a crucial role in *BRCA1/2* carrier BC, supporting the aggressive nature of these tumors and assuming the possible use of novel combination therapy in this subgroup of breast tumor patients [13]. Some studies have also been carried out in order to characterize familial BC that are associated with *BRCA1*/*2* germline mutations, through the evaluation of a panel of different immunohistochemical markers: they showed that *BRCA1* and *BRCA2* tumors can be differentiated because they have a specific immunohistochemical profile with respect to hormonal receptors, cell cycle, apoptosis and basal cell markers [14–16]. Other studies have, instead, been performed in order to characterize a set of immunohistochemical and pathological markers that could help to distinguish the non-*BRCA1/2* familial tumors from the familial cancers carrying these gene mutations, demonstrating the heterogeneity of familial BC [2, 17]. Given this heterogeneity and the variability in the clinical progression of disease, the identification of a set of biomarkers, rather than a single one, seems to be important to predict tumor behavior for the clinical management of patients and to develop new treatment modalities [18, 19].

Using IHC on tissue microarrays (TMAs), we have focused on familial breast tumors in order to analyze the expression of different biomarkers involved in some pathways: progression (NHERF1, TWIST1, Claudin 1), DNA repair mechanisms (BRCT-repeat inhibitor of hTERT expression (BRIT1), SWItch 5 (SWI5), BRCA1 and PARP1), angiogenesis (vascular endothelial growth factor receptor 1 (VEGFR1), VEGF, HIF-1α and MVD), and breast staminal cell markers (CD44 and CD24). We hypothesized the assessment of an immunoprofile, through the unsupervised hierarchical clustering method, able to characterize those tumors with a different biological behavior for a possible future prognostic or therapeutic aim.

## RESULTS

### Protein expression profiling

A cohort of 95 familial BC patients was analysed in this study and their tumor characteristics are shown in Table 1. The frequency of the immunohistochemical expression of NHERF1, TWIST1, Claudin 1, BRIT1, SWI5, BRCA1, PARP1, VEGFR1, VEGF, HIF-1α, MVD, CD44 and CD24 was evaluated on TMAs containing 285 specimens from 95 familial BC patients. Cytoplasmic or nuclear NHERF1 (cNHERF1 and nNHERF1, respectively) expression was evaluated in 84.2% (80/95) of tumor samples. NHERF1 immunostaining was predominantly cytoplasmic, however in some cases an intense nuclear staining was also demonstrated. This was scored separately and its significance was evaluated. cNHERF1 was positive in 55% (44/80) of cases, while nNHERF1 was positive in 13.7% (11/80) of cases. Only two cases were positive for both cNHERF1 and nNHERF1 expression. Nuclear TWIST1 immunostaining was noted in 75.8% (72/95) of analyzed samples; the positive cases were 44.4% (32/72). Claudin 1 membrane immunoreactivity was observed in 81.1% (77/95) of tumor samples, and the positive cases were 28.6% (22/77). BRIT1 showed a cytoplasmic or nuclear staining, which was scored separately, in 72.6% (69/95) of cases. Cytoplasmic BRIT1 (cBRIT1) was positive in 52.2% (36/69) of tumor samples, while nuclear BRIT1 (nBRIT1) showed a positive staining in 44.9% (31/69) of samples. SWI5 showed a cytoplasmic immunoreactivity in 83.2% (79/95) of cases and positive staining was observed in 51.9% (41/79) of samples. BRCA1 immunoreactivity was noted in 91.6% (87/95) of samples and the nuclear-stained positive cases were 41.4% (36/87). Nuclear PARP1 expression was observed in 73.7% (70/95) of familial BCs and was positive in 15.7% (11/70) of samples. VEGFR1 expression was observed in 90.5% (86/95) of cases and showed a mainly cytoplasmic staining. The positive tumor samples constituted 46.5% (40/86) of cases. Cytoplasmic VEGF expression was observed in 85.2% (81/95) of familial tumors and was positive in 70.4% (57/81) of cases. HIF-1α immunoreactivity was observed in 88.4% (84/95) of tumor samples examined. Only cells with completely dark, perinecrotic or diffuse stained nuclei were considered and the positive cases were 33.3% (28/84). MVD was observed in 84.2% (80/95) of samples, and was “high” or positive in 57.5% (46/80) of cases. A predominantly membranous localization was noted as regards CD44 expression, found in 87.4% (83/95) of samples. The positive cases were 49.4% (41/83). Cytoplasmic CD24 staining was observed in 89.5% (85/95) of tumor samples while the positive cases were 71.8% (61/85). In our study we found only six familial BCs with CD44 positive/CD24 negative phenotype that defines cancer stem cells in BC [20]. Given the small case number we did not perform further analyses on this patient subgroup.

**Table 1:** Tumor characteristics of familial breast cancer patients

Characteristics	Familial^a^ (*N* = 95)	
	*N*	(%)
**Age *(median, range)***	45 (24–74)	
≤45	50	(52.6)
>45	45	(47.4)
**Tumor size *(cm)***		
≤2	43	(45.3)
>2	52	(54.7)
**Lymph node status**		
Negative	35	(38)
Positive	57	(62)
**Grade**		
G1 + G2	57	(60)
G3	38	(40)
**Perineoplastic invasion**		
Absent	53	(58.2)
Present	38	(41.8)
**ER**		
Negative	34	(35.8)
Positive	61	(64.2)
**PR**		
Negative	37	(39.4)
Positive	57	(60.6)
**MIB1**		
Negative	31	(32.9)
Positive	63	(67.1)
**HER2**		
Negative	46	(55.4)
Positive	37	(44.6)

a The total number of familial patients considered in this study including the 6 bilateral tumors.

Three patients had missing values for lymph node status, four patients had missing values for perineoplastic invasion, one patient for PR and MIB1 and twelve patients had missing values for HER2.

**Figure 1:**
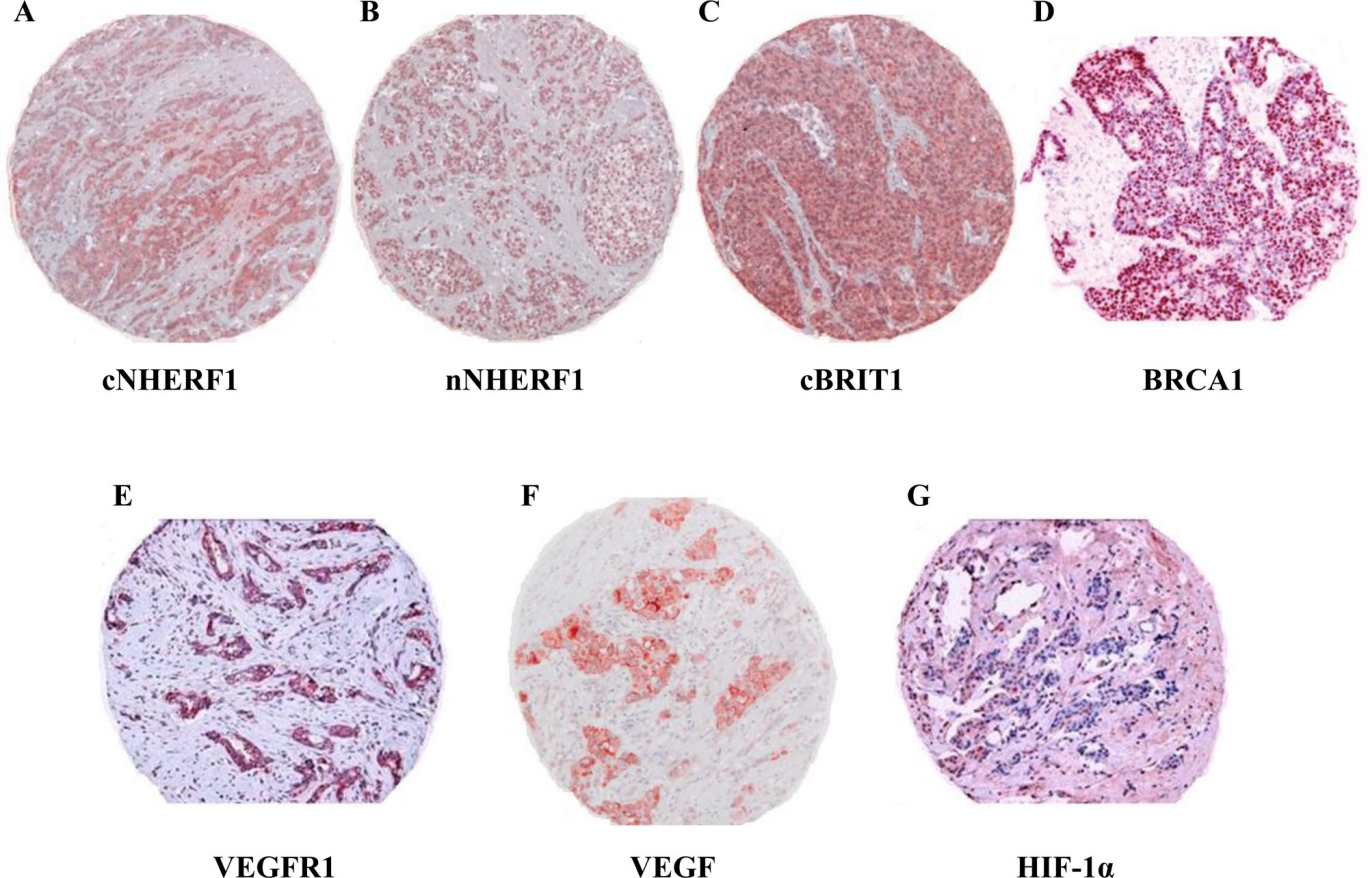
Expression of biomarkers studied by immunohistochemistry on tissue microarrays Representative immunohistochemical staining of a tumor core for the more significant biomarkers identified by statistical analysis. **A.** Cytoplasmic NHERF1 (cNHERF1), **B.** nuclear NHERF1 (nNHERF1), **C.** cytoplasmic BRIT1 (cBRIT1), **D.** nuclear BRCA1, **E.** cytoplasmic VEGFR1, **F.** cytoplasmic VEGF and **G.** nuclear HIF-1α expressions and subcellular localizations. Magnification ×100.

Representative examples of the immunohistochemical staining for the more significant biomarkers on TMA sections, as reported below, are shown in Figure 1 (A-G).

### Analyses of 95 familial breast tumors

**Figure 2:**
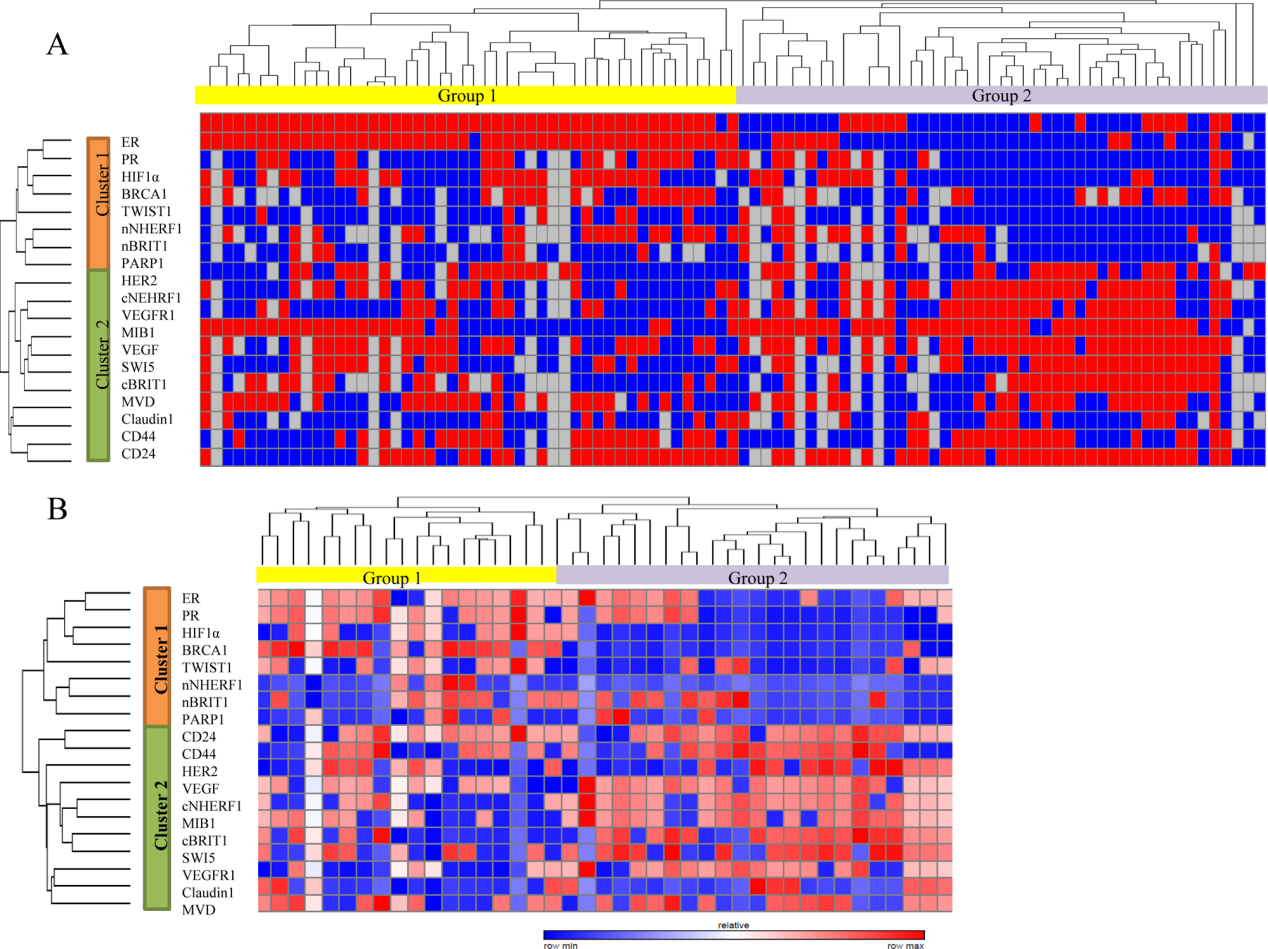
Unsupervised hierarchical analysis based on immunohistochemical score data and survival analysis **A.** Clustergram of 95 familial breast cancer patients over 19 biomarkers. **B.** Clustergram of 45 familial breast tumors over the same biomarkers, which were contemporarily evaluable, excluding missing data.

Unsupervised hierarchical clustering analysis (HCA) was performed on the 19 immunomarkers (cNHERF1, nNHERF1, TWIST1, Claudin 1, cBRIT1, nBRIT1, SWI5, BRCA1, PARP1, VEGFR1, VEGF, HIF-1α, MVD, CD44, CD24, ER, PR, MIB1 and HER2), in order to organize score data into structures based on similarity and dissimilarity of immunostaining profiles. In the first instance, the entire dataset of 95 familial BCs was considered for the analysis, including missing data such as non-evaluable immunomarkers. In Figure 2A, it can be observed that the dendrogram defined two sample clusters (Group 1 and Group 2), characterized by two clusters of biomarkers (Cluster 1 and Cluster 2). In detail, Cluster 1 included ER, PR, HIF-1α, BRCA1, TWIST1, nNHERF1, nBRIT1 and PARP1; Cluster 2 included HER2, cNHERF1, VEGFR1, MIB1, VEGF, SWI5, cBRIT1, MVD, Claudin 1, CD44 and CD24. The Group 1 cluster is characterized by the overexpression of Cluster 1 and the underexpression of Cluster 2, while Group 2 showed an opposite behavior. We analysed the distribution of each biomarker between Group 1 and Group 2 in order to determine which ones contributed to the formation of the two patient groups. We found a statistically significant result only for ER (*P* < 0.001), PR (*P* < 0.001), MIB1 (*P* = 0.002), cNHERF1 (*P* = 0.015), VEGFR1(*P* = 0.001) and BRCA1 (*P* = 0.005), while for VEGF we noticed a trend (*P* = 0.054) (data not shown). These 7 biomarkers were considered for kappa statistics. The overall concordance between assignment of patients to one of the two sample clusters (Group 1 and Group 2) formed when 19 *versus* 7 markers were used showed a substantial agreement (kappa = 0.79). We also analysed the correlations between Group 1 and Group 2 with the clinicopathological characteristics such as tumor size, lymph node status, grade and perineoplastic invasion. A statistically significant result was observed for tumor size (*P* = 0.013) and grade (*P* = 0.001) (data not shown).

### Analyses of 45 familial breast tumors

In order to better define a more precise immunoprofile, we decided to cluster data including only those cases which had all considered biomarkers contemporarily evaluable, obtaining a subgroup of 45 familial BC patients. It was interesting to note that HCA produced the same clusters (Cluster 1 and Cluster 2) of the biomarkers and two sample clusters (Group 1 and Group 2). It is noteworthy that also in this second clustering analysis, Cluster 1 is overexpressed in Group 1 and underexpressed in Group 2, while Cluster 2 is underexpressed in Group 1 and overexpressed in Group 2 (Figure 2B), as described above. When we performed the statistical analysis to show the distribution of each biomarker between Group 1 and Group 2 we found, as previously demonstrated on the 95 cases, a statistically significant correlation for the following biomarkers: ER (*P* = 0.009), PR (*P* < 0.001), MIB1 (*P* = 0.043), cNHERF1 (*P* = 0.004), VEGFR1 (*P* = 0.029) and BRCA1 (*P* = 0.005), but also a significant result was reached for VEGF (*P* = 0.024), nNHERF1 (*P* = 0.026), HIF-1α (*P* < 0.001) and cBRIT1 (*P* = 0.001). The other analysed biomarkers showed no statistically significant distribution (Table 2). The analyses performed on 45 familial breast tumors evidenced an immunoprofile including 10 biomarkers. The overall concordance between assignment of patients to one of the two sample clusters (Group 1 and Group 2) formed when 19 *versus* 10 markers were used showed an almost perfect agreement (kappa = 0.86). When the clinicopatological characteristics were correlated with the two patient groups, identified by this second HCA, we found, as for 95 familial breast tumors, a statistically significant correlation only for tumor size (*P* = 0.012) and grade (*P* = 0.006). Patients belonging to Group 2 had predominantly tumor size >2 cm (80.8%) and were mainly G3 grade (65.4%).

**Table 2:** Correlation of Group 1 and Group 2 patients with biomarker expression in 45 familial breast tumors

Biomarkers	Group 1	Group 2	*P*-value
	*N* (%)	*N* (%)	
**ER**			
Negative	2 (10.5)	13 (50)	0.009
Positive	17 (89.5)	13 (50)	
**PR**			
Negative	2 (10.5)	18 (69.2)	<0.001
Positive	17 (89.5)	8 (30.8)	
**MIB1**			
Negative	9 (47.4)	4 (15.4)	0.043
Positive	10 (52.6)	22 (84.6)	
**HER2**			
Negative	11 (57.9)	15 (57.7)	NS
Positive	8 (42.1)	11 (42.3)	
**cNHERF1**			
Negative	11 (57.9)	4 (15.4)	0.004
Positive	8 (42.1)	22 (84.6)	
**nNHERF1**			
Negative	15 (78.9)	26 (100)	0.026
Positive	4 (21.1)	0 (0)	
**TWIST1**			
Negative	8 (42.1)	16 (61.5)	NS
Positive	11 (57.9)	10 (38.5)	
**Claudin 1**			
Negative	14 (73.7)	20 (76.9)	NS
Positive	5 (26.3)	6 (23.1)	
**cBRIT1**			
Negative	14 (73.7)	5 (19.2)	0.001
Positive	5 (26.3)	21 (80.8)	
**nBRIT1**			
Negative	9 (47.4)	18 (69.2)	NS
Positive	10 (52.6)	8 (30.8)	
**SWI5**			
Negative	10 (52.6)	7 (26.9)	NS
Positive	9 (47.4)	19 (73.1)	
**BRCA1**			
Negative	4 (21.1)	24 (92.3)	<0.001
Positive	15 (78.9)	2 (7.7)	
**PARP1**			
Negative	15 (78.9)	23 (88.5)	NS
Positive	4 (21.1)	3 (11.5)	
**VEGFR1**			
Negative	11 (57.9)	6 (23.1)	0.029
Positive	8 (42.1)	20 (76.9)	
**VEGF**			
Negative	7 (36.8)	2 (7.7)	0.024
Positive	12 (63.2)	24 (92.3)	
**HIF-1α**			
Negative	7 (36.8)	26 (100)	<0.001
Positive	12 (63.2)	0 (0)	
**MVD**			
Negative	8 (42.1)	10 (38.5)	NS
Positive	11 (57.9)	16 (61.5)	
**CD44**			
Negative	9 (47.4)	11 (42.3)	NS
Positive	10 (52.6)	15 (57.7)	
**CD24**			
Negative	3 (15.8)	4 (15.4)	NS
Positive	16 (84.2)	22 (84.6)	

Abbreviations: cNHERF1, cytoplasmic NHERF1; nNHERF1, nuclear NHERF1; cBRIT1, cytoplasmic BRIT1; nBRIT1, nuclear BRIT1; NS, not significant

### Univariate, multivariate and survival analyses

As reported above, univariate analysis was performed for the 10 biomarkers resulting significant, considering the effective evaluable cases for each of them compared to all clinicopathological characteristics. Univariate analysis revealed that negative staining of ER (*P* = 0.007, odds ratio (OR) = 3.49), nNHERF1 (*P* = 0.039, OR = 4.41), HIF-1α (*P* = 0.032, OR = 2.78) and BRCA1 (*P* = 0.035, OR = 2.56) and positive cBRIT1 (*P* = 0.02, OR = 3.11) expression were significantly associated with large tumor size. Negative cNHERF1 expression was found to be associated with positive lymph node status (*P* = 0.016, OR = 3.47). Moreover, negative staining for ER (*P* = 0.0003, OR = 5.15), PR (*P* = 0.006, OR = 3.36), HIF-1α (*P* = 0.0008, OR = 7.44) and BRCA1 (*P* = 0.001, OR = 4.61) together with positive MIB1 (*P* = 0.007, OR = 4.03) and cNHERF1 (*P* = 0.003, OR = 4.53) were significantly related with G3 grade. A statistical trend was found for association between positive cBRIT1 immunostaining and G3 grade (*P* = 0.056, OR = 2.66) (Table 3).

**Table 3:** Univariate analysis

	UNIVARIATE LOGISTIC REGRESSION		
	No. of evaluable cases	OR (95% CI)	*P*-value
**Tumor size >2 *(cm)***			
ER negative	95	3.49 (1.44 ÷ 9.09)	0.007
nNHERF1 negative	80	4.41 (1.16 ÷ 21.52)	0.039
HIF-1α negative	84	2.78 (1.106 ÷ 7.25)	0.032
BRCA1 negative	87	2.56 (1.07 ÷ 6.27)	0.035
cBRIT1 positive	69	3.11 (1.16 ÷ 8.75)	0.025
**Lymph node status positive**			
cNHERF1 negative	80	3.47 (1.29 ÷ 10.21)	0.016
**Grade G3**			
ER negative	95	5.15 (2.12 ÷ 13.13)	0.0003
PR negative	94	3.36 (1.42 ÷ 8.18)	0.006
HIF-1α negative	84	7.44 (2.48 ÷ 27.86)	0.0008
BRCA1 negative	87	4.61 (1.82 ÷ 12.67)	0.001
cBRIT1 positive	69	2.66 (0.99 ÷ 7.54)	0.056
MIB1 positive	94	4.03 (1.53 ÷ 12.08)	0.007
cNHERF1 positive	80	4.53 (1.70 ÷ 13.27)	0.003

Abbreviations: cNHERF1, cytoplasmic NHERF1; nNHERF1, nuclear NHERF1; cBRIT1, cytoplasmic BRIT1; OR, odds ratio; CI, confidence interval

Multivariate analysis was performed on 45 familial breast tumors, considering the 10 significant biomarkers which represented the immunoprofile compared to all clinicopathological characteristics. This analysis identified negative cNHERF1(*P* = 0.005, OR = 12.99) and HIF-1α (*P* = 0.031, OR = 10.28) expression and positive MIB1 (*P* = 0.027, OR = 6.36) staining as independent predictors for positive lymph node status (Table 4).

**Table 4:** Multivariate analysis in 45 familial breast tumors

	MULTIVARIATE LOGISTIC REGRESSION	
	OR (95% CI)	*P*-value
**Lymph node status positive**		
cNHERF1 negative	12.99 (2.48 ÷ 102.3)	0.005
HIF-1α negative	10.28 (1.46 ÷ 111.65)	0.031
MIB1 positive	6.36 (1.36 ÷ 39.55)	0.027

Abbreviations: cNHERF1, cytoplasmic NHERF1; OR, odds ratio; CI, confidence interval

Univariate and multivariate analyses were also performed taking into account the two sample clusters (Group 1 and Group 2) identified through HCA on 45 familial breast tumors. Univariate regression analysis revealed that tumor size >2 cm (*P* = 0.0012, OR = 10.56) and G3 grade (*P* = 0.0023, OR = 13.30) were both associated with Group 2. Interestingly, multivariate analysis confirmed tumor size > 2 cm (*P* = 0.036, OR = 5.95) as an independent variable for the Group 2 sample cluster and showed a trend for G3 grade (*P* = 0.056, OR = 6.20) (Table 5).

**Table 5:** Univariate and multivariate analysis on Group 1 and Group 2 sample cluster in 45 familial breast tumors

	UNIVARIATE LOGISTIC REGRESSION		MULTIVARIATE LOGISTIC REGRESSION	
	OR (95% CI)	*P*-value	OR (95% CI)	*P*-value
**Group 2**				
Tumor size >2 *(cm)*	10.56 (2.70 ÷ 48.90)	0.0012	5.95 (1.15 ÷ 34.79)	0.036
Grade G3	13.30 (2.96 ÷ 96.25)	0.0023	6.20 (1.02 ÷ 51.38)	0.056

Abbreviations: OR, odds ratio; CI, confidence interval

**Figure 3:**
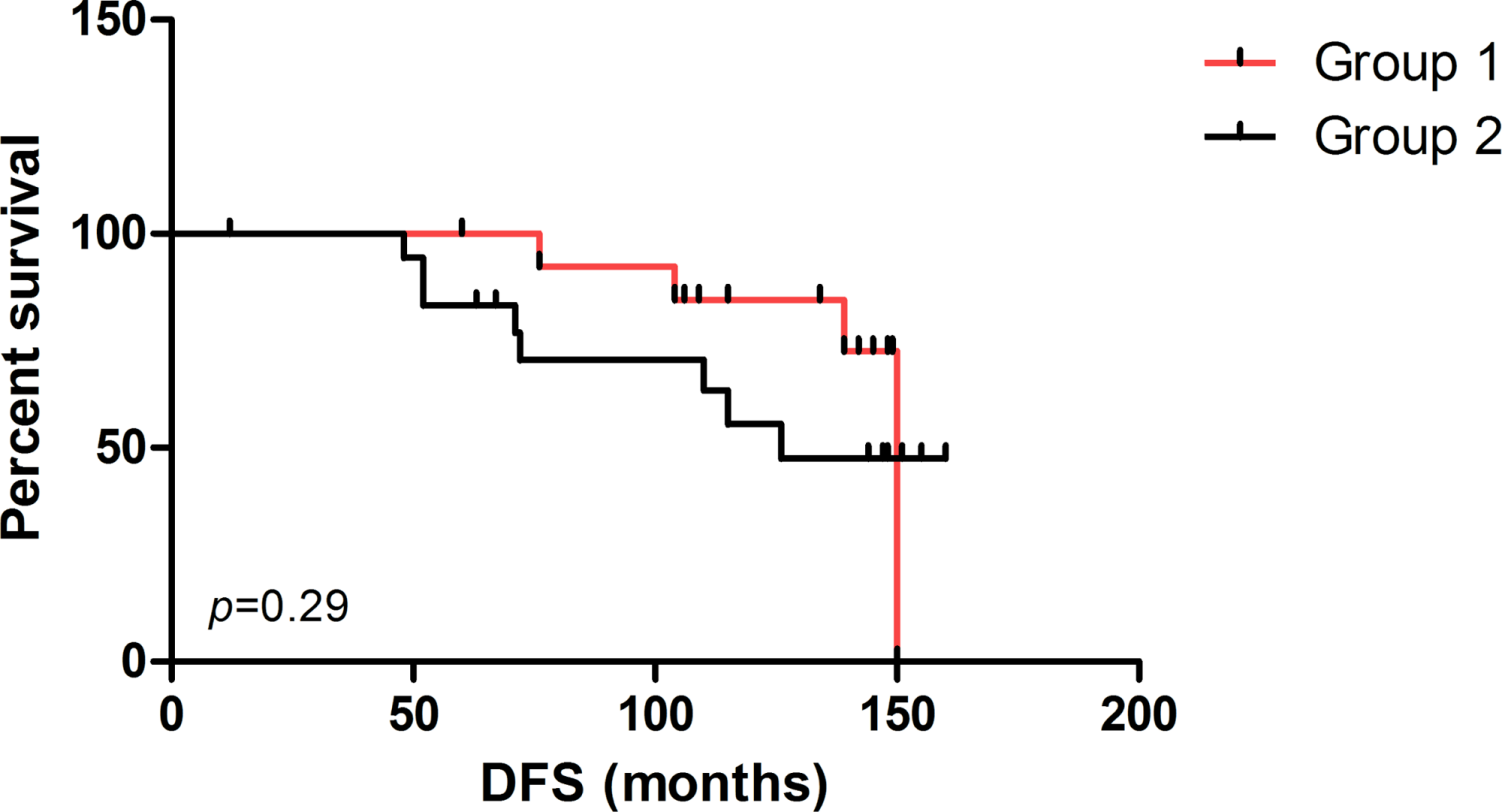
Survival analysis Disease-free survival (DFS) curves for patients in Group 1 and Group 2, based on clustergram including cases with all contemporarily evaluable biomarkers.

The different clinical outcome compared to the disease-free survival (DFS) of Group 2 *versus* Group 1 was revealed by the Kaplan-Meier curve (Figure 3). Complete follow up was available only for 33/45 patients. The median follow up of 33 patients was 115 months. Twelve patients underwent cancer relapse of whom 6 showed distant metastases: in 3 patients these occurred in the boned, in 1 patient in the ovaries, in 1 patient in the cecum, and in 1 patient in the lungs. Although statistical significance was not reached between the two groups, the median DFS of Group 2 was 110 months compared to 137 months in Group 1 patients.

## DISCUSSION

Numerous biomarkers have been proposed as prognostic markers in invasive BC; however, stratification of tumors into prognostic groups to guide therapeutic decision is based mainly on tumor stage and grade and on assessment of ER, PR, MIB1 and HER2 status [6]. The potential for combinations of prognostic markers to be superior to any single marker has been previously observed [18, 19]. Unsupervised HCA based on mRNA levels of thousands of genes has been used to classify breast tumors to identify prognostically relevant cluster groups [21, 22]. However cDNA microarrays, widely used in cancer research, are still far from clinical implementation. In this context, IHC is instead a routinely available method which is also useful to test multiple biomarkers [3]. Some studies, based on HCA of immunomarker data, have been carried out to try to classify in particular sporadic BCs into different cluster groups, but the prognostic significance remained unclear [23, 24]. Makrestov *et al*. [25], on the other hand, demonstrated that by using multiple markers HCA could group breast carcinoma into classes with clinical relevance. Our study is the first to focus only on familial BCs through HCA of the immunohistochemical expression of a set of biomarkers involved in progression, DNA repair and angiogenesis pathways to identify a relevant prognostic immunoprofile.

In the present study, using a panel of 19 biomarkers, unsupervised HCA on 95 familial BCs, and then on the subgroup of 45, identified the same two biomarker clusters (Cluster 1 and Cluster 2) and two groups of patients (Group 1 and Group 2). In detail, Group 2 patients showed an immunoprofile including proteins known to be associated with poor prognosis in sporadic BC. In fact, they were characterized by negativity of ER and PR, HER2 overexpression and increased proliferative activity, which are all correlated with poor outcome [26–28]. Group 2 was also characterized by underexpression of nNHERF1 and overexpression of cNHERF1. We had already demonstrated a significant change in the pattern of cellular NHERF1 distribution from normal to in situ to invasive BC tissue, showing that cNHERF1 staining accumulation could suggest an important role in breast carcinoma development and tumor progression [29]. Moreover, specifically in familial BC, we had reported a significantly higher cNHERF1 expression in ER-negative patients, confirming that cNHERF1 overexpression was associated with aggressive clinical parameters and unfavorable prognosis [30]. More recently we have also shown that cNHERF1 overexpression is significantly associated with negative-PR tumors and with HER2 overexpression, and patients with loss of nNHERF1 and negative-ER were associated with reduced survival [8]. These previous studies confirm the more aggressive Group 2 immunophenotype identified in our analyses, characterized by low ER, PR and nNHERF1 expression (Cluster 1 biomarkers) and high cNHERF1 and HER2 expression (Cluster 2 biomarkers). Other evidence of the poor prognosis of these patients is provided by the overexpression of VEGF, VEGFR1 and MVD, involved in the angiogenesis pathway, and of breast staminal cell markers. This is in agreement with Dales *et al.* [31] who showed that VEGFR1 protein expression identified BC patients with a worse outcome, and suggested its use for evaluating tumor aggressiveness in order to select the best therapeutic approach. Interestingly, we also observed in a previous study that tumors overexpressing cNHERF1 and VEGFR1 revealed an association with poor outcome, also characterized by an increasing tumor grade and negative status of steroid hormone receptors [32]. In addition, in familial BC we have found that high VEGF expression is significantly associated with poor tumor grade, MIB1 positive expression and negative ER and PR status. This confirms the close relationship with cancer progression [13], in agreement also with Zhang *et al.* [24]. As reported, Group 2 was characterized by MVD overexpression, and this marker has been already identified as an independent prognostic indicator of recurrence and death for BC [33]. Overexpression of CD24 and CD44 have been correlated also to the malignant transformation and progression of BC, showing an increasing expression in invasive ductal carcinoma compared with ductal carcinoma in situ and intraductal hyperplasia, suggesting that these biomarkers might play an important role in BC development [34]. Group 2 patients are characterized also by BRCA1 underexpression, and this aspect further underlines the aggressiveness of this group. This is supported by our previous study on familial BC in which we found that loss or reduction of both BRCA1 and ER expression were correlated with higher histological grade and lower PR positive status [35], as also demonstrated by Taylor *et al*. [36]. These authors underlined the role of nuclear BRCA1 as a tumor suppressor in BC, and its underexpression might be correlated with a more invasive tumor phenotype [36]. Moreover, Jarvis *et al*. [11] found that the absence of nuclear BRCA1 is significantly associated with the expression of high levels of the proliferation marker, highlighting a more aggressive tumor behavior. Group 2 showed also underexpression of nBRIT1 and overexpression of cBRIT1, and this profile underlines the aggressiveness of this patient subgroup as confirmed by other authors [37, 38]. Interestingly only cytoplasmic staining was detected in high grade tumors. A significant correlation was found between low nBRIT1 expression and high tumor grade and also BRCA1 underexpression by Richardson *et al*. [37], while more recently Jo *et al*. [38] showed that high cBRIT1 expression was significantly associated with high tumor grade. This evidence underlines the poor patient outcome and the more aggressive nature of these tumors, rendering BRIT1 a promising new prognostic biomarker in BC [37, 38]. Interestingly, in Group 2 we found 8 of 11 cases with *BRCA1/2* mutations clustered. This is in agreement with the evidence that carcinomas linked to these gene mutations are proliferating tumors [39], often present higher histological grade [40] and have a low ER/PR positive rate [41]. HCA on 45 familial breast tumors, with all biomarkers contemporarily evaluable, together with the analysis of marker distributions between patient clusters, allowed us to identify a minimal set of 10 significant biomarkers necessary to define more precisely the patient cluster groups. In particular, the immunoprofile characterized by the low expression of ER, PR, BRCA1, nNHERF1 and HIF-1α and the higher expression of MIB1, cNHERF1, cBRIT1, VEGF and VEGFR1 identified familial BC patients with a more aggressive immunophenotype as discussed above. Considering the clinicopathological characteristics, we found that Group 2, as confirmed by univariate and multivariate analyses, was significantly associated with large tumor size and high grade, supporting the evidence of a more aggressive phenotype. A similar study, performed by Honrado *et al.* [17] on non-*BRCA1/2* BC families identified a cluster group characterized by higher grade, ER negativity and the expression of proteins related to proliferation and cell cycle progression, confirming thus the heterogeneity of familial BC. Taking into account the set of 10 significant biomarkers, univariate analysis confirmed that many of these are significantly associated with large tumor size and poorly differentiated tumors. Furthermore, we observed that negative cNHERF1 expression was significantly associated with positive lymph node status. This result, although confirmed by multivariate analysis, differs from the previous evidence, and could be due to the almost homogenous distribution of the lymph node status (positive *versus* negative) between Group 1 and Group 2. Negative nuclear HIF-1α and positive MIB1 expression resulted, by multivariate analysis, independent predictors for positive lymph node status from the other clinicopathological characteristics. The literature reports that nuclear HIF-1α is overexpressed during sporadic breast carcinogenesis and correlated with poor prognosis [42], showing also a more frequent overexpression in BRCA1 related BCs, as previously described by ourselves and others [13, 43]. However, when we analysed the nuclear HIF-1α expression in familial compared to sporadic cancers, we did not observe a substantial difference [30]. The low presence of *BRCA1* mutated cancer in our study and the different antibody used are possible reasons for discrepancies between these results. The correlation between positive proliferative activity and positive lymph node status, instead, confirms the value of MIB1 as a poor independent factor. The same was true for Tan *et al.* [44]. Unfortunately, in the 45 familial breast tumor subgroup, the number of patients with a known and complete follow up was not high enough to determine if there was a statistically significant difference between Group 1 and Group 2 compared to DFS. However, we found that 8 of 12 patients (66.7%) who had reported recurrence were clustered in Group 2, which had also a lower median DFS than Group 1. This feature further underlines the more aggressive Group 2 phenotype referred to the worse clinical outcome.

Our study confirms the phenotypic and clinical heterogeneity of familial BCs. Clinical application of HCA of immunohistochemical data in these cases could allow the assessment of prognostic biomarkers to be used simultaneously. The 10 protein expression panel, found in our study, might be used to identify the more aggressive phenotype of familial breast tumors, and to classify patients into different prognostic groups in order to direct them towards alternative clinical therapies. Validation of this approach will require testing a sufficiently large sample series to allow analysis of familial BC patients with unclear prognosis.

## MATERIALS AND METHODS

### Patients

Ninety five familial breast tumors, arranged in TMA sections, were retrospectively collected for this study. Almost all patients, of whom 3 were male, presented operable infiltrating ductal breast carcinoma and were subjected to primary surgery with nodal dissection at our Institute *IRCCS Istituto Tumori “G. Paolo II”* of Bari, Italy, in the years 2002–2003. The median age was 45 years (range 24–74 years), and six patients had bilateral breast tumors. All patients were classified as “familial”, and screened for *BRCA1*/*2* mutations, due to a familial history of BC and the fact that during genetic counselling one of the following conditions was found: (1) at least 3 relatives (first or second degree) had breast or ovarian cancer; (2) 2 relatives younger than 50 years had BC; (3) 1 relative younger than 36 years had BC; (4) the patient had bilateral cancer and at least 1 relative with BC (or a relative with bilateral cancer); and (5) 1 male patient with BC [45]. All patients gave informed consent to utilize their removed biological tissue for molecular analyses and research purposes, according to ethical standards. The study has been approved by the Ethics Committee of Istituto Tumori “Giovanni Paolo II” of Bari with the reference number 56/CE signed in the 16th of May 2011. Only 11 of the 95 familial cases presented *BRCA1*/*2*–mutated BC, according to full-length gene sequencing analyses. Tumor characteristics, including tumor size, lymph node status, grade, perineoplastic invasion, ER, PR, proliferative activity and HER2 status, were provided by the Pathology Department of our Institute (Table 1). Tumors with ER or PR expression were scored as positive when nuclear staining was present in >10%. For proliferative activity, assessed by MIB1 nuclear staining, we adopted the cut off value of 20% positive cells and the tumours with MIB1 >20% were considered highly proliferating. This cut off represents the median value of the scores relative to all breast tumor samples analysed during the last 5 years within our Institute. HER2 protein expression was investigated using a monoclonal antibody (MoAb clone CB11; Novocastra Laboratories, Ltd., Newcastle, UK) and scored in accordance with the HercepTest scoring system (Food and Drug Administration accepted): 0, no membranous immunoreactivity or <10% of cells reactive; 1+, incomplete membranous reactivity in >10% of cells; 2+, ≥10% of cells with weak to moderate complete membranous reactivity; and 3+, strong and complete membranous reactivity in >10% of cells. Cytoplasmic immunoreactivity was ignored. Cases scoring 0 and 1+ were classified as negative. HER2 was considered to be positive if immunostaining was 3+ or if a score 2+ showed gene amplification by fluorescence in situ hybridization (FISH). In FISH analyses, each copy of the *HER2* gene and its centromere 17 (*CEP17*) reference were counted. The interpretation followed the criteria of the 2007 ASCO/CAP guidelines for HER2 testing in BC [46], positive if the *HER2/CEP17* ratio was higher than 2.2.

### TMAs and IHC

TMAs were assembled from formalin-fixed and paraffin-embedded tissues as previously described [30]. Briefly, three core specimens with a diameter of 0.5 mm were punched from the representative tumor regions of each donor block and were precisely arrayed into new recipient paraffin blocks using a Tissue Microarrayer (Beecker Instruments, Silver Spring, MD, USA). Each sample was arrayed in triplicate to minimize tissue loss and to overcome tumor heterogeneity. The three cores were representative of the whole specimen. All immunoreactivity samples were scored by double-blinded independent observers who had no patient information, and the mean of the three readings for each patient was calculated. If one core was uninformative, either lost or contained no tumor tissue, the overall score applied was that of the remaining cores. The results from the two observers were identical in most cases, and discrepancies were resolved by re-examination and consensus.

All specimens were cut into 4-μm-thick slices to make sections for immunohistochemical staining using standard immunoperoxidase techniques [35]. In brief, TMA slides were deparaffinized and partially rehydrated through absolute ethanol and 95% ethanol series. Antigen retrieval was performed by the 0.01 M citrate buffer (pH 6.0) at 98°C in a water bath from a minimum of 20 to a maximum of 45 minutes, except for the anti-Claudin 1 antibody which requires antigen retrieval by the Tris/EDTA buffer (pH 8.0) at 98°C in a water bath for 45 minutes. The slides were then allowed to cool for 30 minutes and the endogenous peroxidase activity was blocked for 10 minutes with 3% H_2_O_2_. The primary antibodies, diluted in phosphate buffered saline/bovine serum albumin (PBS/BSA) 1%, were incubated on the slides at 4°C overnight in a moist chamber. For anti-VEGFR1, 1 hour incubation at room temperature was required. A polymer-based IHC detection system was used as the amplification system (EnVision + System-HRP Labelled Polymer Anti-Rabbit or Anti-Mouse secondary antibody, Dako, Carpinteria, CA, USA) according to the manufacture's instruction. The bound antibody was visualized by incubating the sections in 3-amino-9-ethylcarbazole (AEC + Substrate Chromogen, Dako, Carpinteria, CA, USA) for 15 minutes, except for anti -CD34, -VEGFR1, -BRCA1, -PARP1 and -Claudin 1 which require the use of 3,3′-diaminobenzidine (Liquid DAB + Substrate Chromogen System, Dako, Carpinteria, CA, USA) for 8–10 minutes. Cell nuclei were counterstained with Mayer's Haematoxylin (Bio-Optica, MI, Italy) and the slides were mounted with aqueous mounting medium (Faramount Aqueous Mounting Medium, Dako, Carpinteria, CA, USA). Known positive controls and a negative control, replacing the primary antibody with PBS1X (pH7.6), were included in each staining run.

Table 6 shows the different analyzed biomarkers, dilution, source/clone, the staining localization of antibody and the cut off [median value, immunohistochemical score (IHS) or quickscore method (QS)] used to classify positive *versus* negative cases. The median value of immunoreactive cells was used as cut off for cNHERF1 (≥40%), nNHERF1 (>0%), TWIST1 (>0%), Claudin 1 (>0%), cBRIT1 (≥17%), nBRIT1 (>0%), SWI5 (≥7%), BRCA1(>0%), VEGFR1 (>0%), HIF-1α (>0%) and MVD (≥14%). For VEGF the HIS was calculated by combining the quantity score (percentage of positive stained cells) with the staining intensity score [13]. The quantity score ranges from 0 to 4: 0 = no immunoreactivity; 1 ≤ 25% cells stained; 2 = 26–50% cells stained; 3 = 51–75% cells stained; and 4 = ≥76% cells stained. The staining intensity was scored as: 0 (negative), 1 (weak), 2 (moderate) and 3 (strong). Raw data were converted to IHS by adding the quantity score (0–4) to the staining intensity score (0–3). Theoretically, the scores can range from 0 to 7. An IHS of 6–7 was considered a strong immunoreactivity; 3–5, moderate; 1–2, weak; and 0, negative. For our analyses, tumors presenting a moderate or strong score were VEGF positive (IHS:3–7). PARP1 immunoreactivity was scored by the multiplicative QS [10, 47]. This system accounts for both the intensity and the extent of cell staining. The proportion of positive cells was estimated and given a score on a scale from 1 to 6 (1 = 1% to 4%, 2 = 5% to 19%, 3 = 20% to 39%, 4 = 40% to 59%, 5 = 60% to 79%, and 6 = 80% to 100%). The average intensity of the positive staining of cells was given a score from 0 to 3 (0 = no staining, 1 = weak, 2 = intermediate, and 3 = strong staining). A final score was calculated by multiplying the percentage score by the intensity score. Based on the final score, PARP1 expression was graded as negative (0–9) or positive (10–18). The tumor was considered positive for CD44 and CD24 biomarkers when a moderate to strong staining was observed in more than 10% (cut off) of tumor cells, as *per* previous publications [48, 49]. Finally, microvessel counting was performed by identifying the areas which represented the highest vascular density - so called “hot spots”. The MVD measures were made in the fields with a higher density of CD34 positive cells and cell clusters at 200× magnification, as previously described [13].

**Table 6:** Dilution, source, staining of antibodies and cut off used

Biomarkers	Dilution	Source/clone	Staining localization	Cut off (range)
*Progression biomarkers*				
NHERF1	1:150	Affinity Bioreagents, rabbit polyclonal EBP50, PA1-090	cytoplasmic	≥40%* (10–70%)
			nuclear	>0%* (0–40%)
TWIST1	1:50	Abcam, mouse monoclonal, Twist2C1a	nuclear	>0%* (0–69%)
Claudin 1	1:25	Invitrogen, rabbit polyclonal, JAY.8	membranous	>0%* (0–70%)
*DNA repair mechanism biomarkers*				
BRIT1	1:100	Abcam, rabbit polyclonal	cytoplasmic	≥17%* (0–80%)
			nuclear	>0%* (0–70%)
SWI5	1:150	Santa Cruz, rabbit polyclonal, C-13	cytoplasmic	≥7%* (0–80%)
BRCA1	1:75	Oncogene Research, mouse monoclonal, MS110	nuclear	>0%* (0–55%)
PARP1	1:100	Santa Cruz, mouse monoclonal, F-2	nuclear	≥10** (0–18)
*Angiogenesis biomarkers*				
VEGFR1	1:100	Santa Cruz, rabbit polyclonal Flt1, C-17	cytoplasmic	>0%* (0–80%)
VEGF	1:50	Santa Cruz, rabbit polyclonal, A-20	cytoplasmic	≥3^***^ (0–7)
HIF-1α	1:50	Santa Cruz, rabbit polyclonal, H-206	nuclear	>0%* (0–50%)
MVD	1:50	Novocastra, anti-CD34, mouse monoclonal, QBEnd/10		≥14%* (5–30%)
*Breast staminal cell biomarkers*				
CD44	1:150	Dako, mouse monoclonal, DF1485	membranous	>10%
CD24	1:100	Millipore, mouse monoclonal, SN3	cytoplasmic	>10%

* median value;

** quickscore method;

*** immunohistochemical score

### Hierarchical clustering and statistical analyses

Unsupervised HCA was performed using the immunohistochemical score data of each biomarker through the same approach adopted for cDNA microarray data [50].

The java-based tool GENE-E (http://www.broadinstitute.org/cancer/software/GENE-E/index.html) was used to carry out clustering, merging objects based on their pair-wise distance. The average linkage method was used to obtain cluster dendrograms both for biomarkers and cases, which could be seen respectively on the left side and top of the heatmap. A strong positive score is represented by a red block and a negative score appears as a blue block. Non-evaluable stains are represented by grey blocks. The Chi-square (χ^2^) Fisher's exact test was assessed in order to evaluate the correlations between the two groups of patients (Group 1 and Group 2), identified by HCA, with the clinicopathological tumor characteristics (tumor size, lymph node status, grade and perineoplastic invasion), and to determine which biomarkers contributed to the formation of cluster groups. Statistical analyses were performed using SPSS 17.0 software (SPSS, Inc, Chicago, IL, USA). Agreement in the classification of cases based on hierarchical clustering was assessed with kappa statistics. A kappa value of 0.41 to 0.6 indicates moderate agreement, 0.61 to 0.8 substantial agreement and more than 0.8 almost perfect agreement [51]. Kappa statistics were carried out through R package “irr” [52]. Univariate and multivariate analyses were carried out to correlate immunomarkers with clinicopathological features. A generalized linear model was fitted through the glm() function of R package “MASS”. The clustered patient groups were assessed in relation to DFS. DFS (in months) was defined as the time from diagnosis to the date of locoregional or distant recurrence, second invasive breast carcinoma, second primary cancer without evidence of BC or to the date of last contact. DFS probability of the clustered patient groups was computed by the Kaplan-Meier product limit method and compared by the log rank test. Survival analysis was performed through GraphPad Prism 5.0.1. Results from all statistical analyses were considered to be significant at a level of *P*-values less than 0.05.
